# Relationship of tooth loss to mild memory impairment and cognitive impairment: findings from the fujiwara-kyo study

**DOI:** 10.1186/1744-9081-6-77

**Published:** 2010-12-31

**Authors:** Nozomi Okamoto, Masayuki Morikawa, Kensuke Okamoto, Noboru Habu, Junko Iwamoto, Kimiko Tomioka, Keigo Saeki, Motokazu Yanagi, Nobuko Amano, Norio Kurumatani

**Affiliations:** 1 Department of Community Health and Epidemiology, Nara Medical University, Japan; 2 Sakai City Mental Health Center, Japan; 3 Department of Psychiatry, Nara Medical University, Japan; 4 Department of Oral and Maxillofacial Surgery, Nara Kasuga Hospital, Japan; 5 Department of Oral and Maxillofacial Surgery, Nara Medical University, Japan; 6 Department of Indoor Environmental Medicine, Nara Medical University, Japan; 7 Department of Food and Nutrition, Tezukayama University, Japan

## Abstract

**Background:**

This cross-sectional study investigated the relationship between the number of remaining teeth to mild memory impairment (MMI), which is a preclinical stage of dementia, and to cognitive impairment.

**Methods:**

The subjects were aged 65 years or older and were grouped according to their score for the Mini-Mental State Examination (MMSE), the three-word delayed recall test in the MMSE, and the Geriatric Depression Scale into the control group (n = 3,696), the MMI group (n = 121), and the low MMSE score (23 or lower) group (n = 214). We collected data on the number of remaining teeth, the length of the edentulous period, health-related lifestyle, medical history, blood pressure, height, and body weight. Fasting venous blood samples were also obtained.

**Results:**

Multiple logistic regression analysis, adjusted for depressive symptoms, age, sex, length of education, and other explanatory variables, revealed that the odds ratios of 0-10 remaining teeth to 22-32 remaining teeth were 1.679 (95% CI 1.073-2.627) for MMI and 2.177 (95% CI 1.510-3.140) for a low MMSE score. A significant relationship was also found between the length of the edentulous period and the risk of a low MMSE score (odds ratio 3.102, 95% CI 1.432-6.720) (15 years or more/less than 15 years).

**Conclusions:**

Our findings suggest that tooth loss is associated with cognitive function.

## Background

Tooth loss, one of the indicators of periodontal disease [1,2], has been reported to be associated with Alzheimer's disease (AD) and dementia [3,4]. Individuals with clinical dementia have an increased deterioration of their dental health [5], and tooth loss may induce nutritional deficits [6]. Reductions in the number of pyramidal cells [7] and acetylcholine levels [8] in the hippocampus due to the decrease of masticatory function caused by molar loss have been found in animal models. Furthermore, it has been hypothesized [9] that periodontal disease-derived inflammatory molecules, bacteria, and bacterial products enhance brain inflammation [10,11].

Among individuals with mild memory impairment (MMI), 21.2% progressed to illnesses with dementia, including AD (10.6%), vascular dementia (4.8%), or other types of dementia (5.8%), over a period of 5 years [12]; therefore, they represent a high-risk population for dementia. MMI was defined as [13]: (1) no impairment of the activities of daily living (ADL); (2) normal general cognitive function, as determined by a Mini-Mental State Examination (MMSE) score ≥ 24 [14]; (3) objective memory impairment, assessed by the MMSE three-word delayed recall test (Recall test) (low score: 1 or 0); and (4) absence of dementia or depression, diagnosed by geriatric neuropsychiatrists according to the Diagnostic and Statistical Manual of Mental Disorders, 3^rd ^edn., revised (DSM-III R) criteria [15].

We hypothesized that tooth loss may also be associated with the preclinical stage of AD and dementia. To investigate our hypothesis in a community-based survey, subjects with MMI or a low MMSE score (23 or lower) [16] and elderly controls were identified operationally by using the MMSE and the Geriatric Depression Scale (GDS) short version [17]. Data on the number of remaining teeth, the length of the edentulous period, health-related lifestyles, and medical history were collected. Blood pressure, height, and body weight were measured, and fasting venous blood samples were also obtained. The purpose of this cross-sectional study was to compare the number of remaining teeth and the length of the edentulous period in subjects with MMI or a low MMSE score with those in elderly controls.

## Methods

### 1. Subjects

This study was approved by the Medical Ethics Committee of Nara Medical University. Written informed consent was obtained from each of the subjects prior to their participation in the study.

We used data from the baseline examination of the Fujiwara-kyo study, a study of successful aging in the elderly. The subjects were volunteer men and women aged 65 years or older from Nara prefecture (where the first capital of Japan, called "Fujiwara-kyo," was established), who were living in their own homes and able to walk independently. A total of 4,206 individuals gave their written consent for participation in the study and completed the baseline examination in 2007-2008.

### 2. Assessment of cognitive mental status

The MMSE (score range, 0-30) is used as a screening test for cognitive impairment. The Recall test (score range, 0-3) is a sub-item of the MMSE that evaluates the impairment of recent memory. Subjects were instructed to recall three unrelated objects that they were previously instructed to remember. The MMSE was carried out by clinical psychologists or interviewers who majored in psychology in graduate schools and were formally trained by a psychiatrist.

Depression was evaluated using the GDS (score range, 0-15; cut-off score, 5/6). Responses were coded as follows: 1 = yes, symptom present; 0 = no, symptom not present. The scores for the individual items were summed to obtain the final score, where higher scores indicated a greater number of depressive symptoms. The GDS was included in a self-administered questionnaire survey.

### 3. Dental examination

A dental examination was carried out by two dentists calibrated as to the techniques, with the dentist and the subject in a sitting position under artificial lighting. The number of teeth was recorded for each subject. The remaining teeth were defined as healthy, carious, or treated teeth (including crowned, inlay, and abutment teeth for bridge work), inclusive of completely erupted third molars. Root tips and very loose teeth that were indicated for extraction were not included as remaining teeth. The age at which edentulous individuals had lost all of their teeth was also recorded. The Community Periodontal Index (CPI) code [18] was recorded. The upper and lower teeth were divided into six prescribed segments, and each segment was subjected to further examination if it contained two or more remaining teeth that did not require extraction. The prescribed 10 representative teeth in the 6 segments (tooth position: 11, 16, 17, 26, 27, 31, 36, 37, 46, and 47) were examined. Each segment was assigned to one of five code levels (code 0, healthy; code 1, gingival bleeding after probing; code 2, calculus present in the periodontal pocket; code 3, periodontal pocket 4-5 mm deep; and code 4, periodontal pocket at least 6 mm deep), or an ineligible segment (a segment with one or no remaining teeth). The highest code level among the six segments was regarded as the maximum CPI code for the individual.

### 4. Other recorded variables

The subjects were asked about their drinking frequency (hardly drink; drink on at least 1 day a week), the type of alcohol they drank, their average daily alcohol intake, their smoking habits (never; ex-smoker; current smoker), and the time spent walking every day on average (less than 30 minutes; 30 minutes or more) in a self-administered questionnaire that was confirmed by interview. Each subject was interviewed to record the presence or absence of severe visual and hearing impairment, impairment of the basic (eating, dressing, bathing, toileting, and walking) and instrumental (use public transportation, shop for daily necessities, pay bills, and handle one's own banking) ADL, and any history of disease (cancer, cerebrovascular disease, myocardial infarction, diabetes mellitus, and hypertension). After sitting quietly for more than 5 min, their blood pressure was determined twice at an interval of 30 s using an automatic blood-pressure manometer (ES-P2100; TERUMO Co., Tokyo, Japan) that displayed the values determined by the Korotkoff method. An average of two measurements was used in the analyses. The height and body weight of the subjects were measured using a body fat scale (TBF-215; TANITA Co., Tokyo, Japan) with the subject wearing an examination gown. Body mass index was calculated as weight (kg) divided by height squared (m^2^). Fasting venous blood samples were drawn (serum albumin, hemoglobin A1c, total cholesterol, and low-density lipoprotein cholesterol). These examinations were carried out by medical professionals.

### 5. Statistical analysis

Examinations of the differences between the control and MMI groups, and between the control and low MMSE score groups were performed at the level of significance set for multiple comparisons based on the Bonferroni test, after the chi-square and Mann-Whitney tests were used. A trend test to detect the increased prevalence of MMI or a low MMSE score status according to the number of remaining teeth and CPI was performed using the Mantel-extension method [19]. Logistic regression analysis (by the forced entry method) was carried out with MMI and a low MMSE score as dependent variables. Depressive symptoms, sex, age, length of education, and other variables were used as independent variables. The number of remaining teeth and the length of time after the subjects became edentulous were used as continuous variables in one model and categorical variables in the other model. Goodness of fit was performed based on the technique of Hosmer and Lemeshow [20]. Statistical analysis was performed using SPSS (version 17.0; SPSS Japan Inc., Tokyo, Japan). We calculated two-tailed P values in all of the analyses. The α level of significance was set at 0.05.

## Results

**Figure 1:**
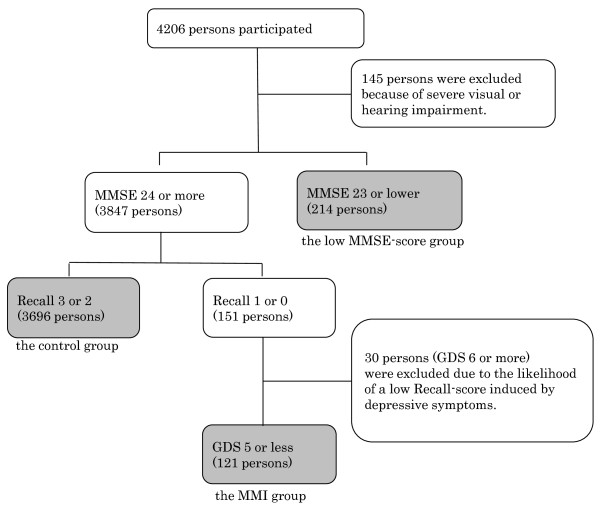
**Selection of subjects**. MMI: Mild Memory Impairment, MMSE: Mini-Mental State Examination, GDS: Geriatric Depression Scale.

Figure 1 shows the procedure for selecting the subjects. Among the 4,206 participants examined, we excluded 145 individuals who were revealed by interviews to have severe visual or hearing impairment that were likely to affect the cognitive function tests. Of the 4,061 remaining subjects, 214 had low MMSE scores (23 or lower). The 3,847 subjects with high MMSE scores (24 or more) were revealed by interviews to be able to independently execute the basic and instrumental ADL. They were classified on the basis of their Recall score into the control group (high score: 3 or 2) (n = 3,696) or the low score group (score: 1 or 0) (n = 151). We excluded 30 subjects from the low score group who were suspected of having depression based on their high scores in the GDS (score: 6 or more) due to the likelihood of pseudo-dementia induced by depressive symptoms. Finally, the control (n = 3,696), MMI (n = 121), and low MMSE score (n = 214) groups were included for analysis in this cross-sectional study.

Table 1 shows the demographic characteristics of the subjects. The factors showing statistically significant differences by the Bonferroni test between the control and MMI groups or between the control and low MMSE score groups were depressive symptoms, age, sex, length of education, the number of remaining teeth, drinking alcohol at least one day a week, smoking, time spent walking every day, and the levels of serum albumin, total cholesterol, and low-density lipoprotein cholesterol.

**Table 1:** Demographic characteristics of the subjects

Variables	Control n = 3696	MMI n = 121	Low MMSE score n = 214	**Control vs**. MMI P value*	Control vs. Low MMSE score P value*
MMSE-total	28.0 (26.0, 30.0)	26.0 (25.0, 27.0)	23.0 (22.0, 23.0)	<0.001	<0.001
Depressive symptoms					
GDS 6 or more	14.4%	--†	23.8%	--†	<0.001
Age (y)	71.0 (68.0, 75.0)	74.0 (71.0, 79.0)	74.0 (71.0, 78.0)	<0.001	<0.001
Female	51.7%	31.4%	42.5%	<0.001	0.009
Length of education					
Less than 12 years	26.6%	37.2%	63.6%	0.012	<0.001
Oral health					
Number of remaining teeth	21.0 (11.0, 26.0)	16.0 (4.0, 25.0)	12.5 (1.8, 22.0)	0.001	<0.001
CPI					
Ineligible‡	13.2%	23.1%	27.6%		
Code 0, 1, or 2	26.3%	18.1%	19.1%		
Code 3	38.3%	31.4%	34.1%	0.082	0.577
Code 4	22.2%	27.3%	19.2%		
Drinking habits					
At least 1 day a weak	38.1%	51.2%	38.3%	0.004	0.961
Ethanol intake 40 g or more per day	28.9%	25.5%	24.2%	0.752	0.473
Smoking habit					
Never	59.0%	42.9%	54.2%		
Ex-smoker	31.8%	47.9%	29.9%	<0.001	<0.001
Current smoker	9.2%	9.1%	15.9%		
Time spent walking every day on average					
Less than 30 minutes	8.0%	9.9%	13.1%	0.396	0.014
Positive history of disease					
Cancer	9.1%	14.9%	10.7%	0.037	0.394
Cerebrovascular disease	5.7%	9.1%	6.5%	0.117	0.650
Myocardial infarction	2.6%	2.5%	2.3%	0.936	0.815
Diabetes mellitus	10.8%	13.2%	15.4%	0.375	0.043
Hypertension	39.1%	38.8%	41.6%	0.960	0.472
Medical examination					
Systolic blood pressure (mmHg)	142 (129, 155)	146 (128, 159)	145 (132, 159)	0.350	0.080
Diastolic blood pressure (mmHg)	76 (68, 83)	75 (66, 84)	75 (66, 83)	0.256	0.121
Body mass index	22.8 (20.9, 24.7)	22.9 (20.8, 24.6)	22.9 (20.8, 25.6)	0.761	0.581
Serum albumin (g/dL)	4.5 (4.4, 4.7)	4.5 (4.3, 4.6)	4.6 (4.3, 4.7)	0.002	0.758
Hemogrobin A1c (%)	5.1 (4.9, 5.5)	5.1 (4.8, 5.5)	5.2 (4.9, 5.5)	0.484	0.978
Total cholesterol (mg/dL)	215.0 (193.0, 237.0)	205.5 (186.0, 228.0)	202.5 (181.8, 232.3)	0.011	<0.001
Low-density lipoprotein cholesterol (mg/dL)	126.0 (107.0, 147.0)	123.0 (102.0, 140.5)	120.0 (98.8, 139.3)	0.065	0.003

Data are given as % or median (25th percentile,75th percentile).

MMI: Mild Memory Impairment, MMSE: Mini-Mental State Examination, GDS: Geriatric Depression Scale, CPI: Community Periodontal Index.

*The chi-square test or Mann-Whitney test was performed.

†The subjects whose GDS score were "6 or more" were excluded from the MMI group.

‡Subjects in whom none of the six fractions could be evaluated because of the low number of remaining teeth.

**Figure 2:**
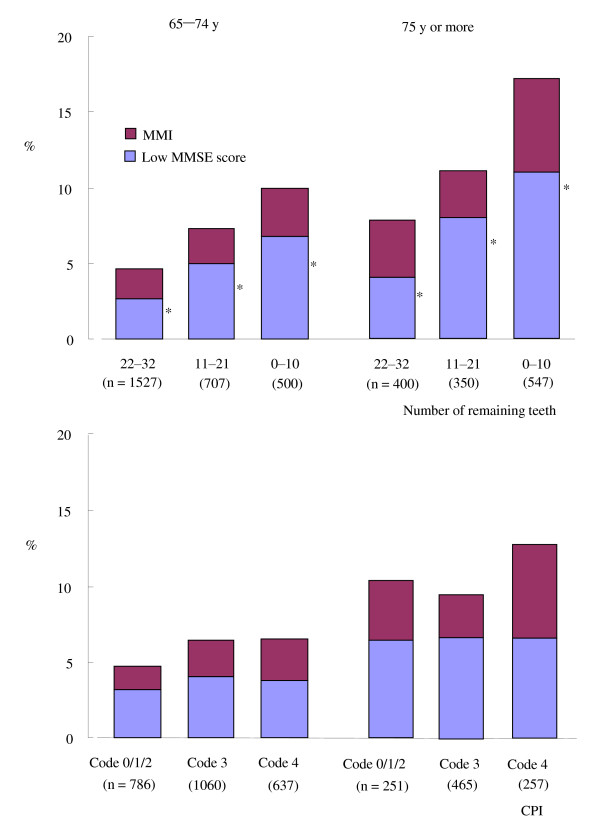
**Prevalence of MMI or low MMSE-score according to dental status**. MMI: Mild Memory Impairment, MMSE: Mini-Mental State Examination, CPI: Community Periodontal Index. *P < 0.001. A trend test to detect the increased prevalence was performed using the Mantel-extension method.

Figure 2 shows the prevalence of MMI or a low MMSE score. The number of remaining teeth (range: 0-32) was categorized into 3 equal categories (22-32, 11-21, and 0-10 teeth). No significant dose-response relationship was found in a trend test between the number of remaining teeth and the prevalence of MMI in the 65-74 years or 75 years or more categories, although the prevalence of MMI was higher in subjects with 0-10 remaining teeth than in those with 22-32 remaining teeth. The prevalence of a low MMSE score was 2.7% in subjects with 22-32 remaining teeth, 5.0% in those with 11-21 remaining teeth, and 6.8% in those with 0-10 remaining teeth in the 65-74 years category, and 4.0%, 8.0%, and 11.0% in the 75 years or more category, respectively. Significant increases in prevalence were found in a trend test in both age categories (P < 0.001) (upper panel, Figure 2). There was no significant dose-response relationship in a trend test between the CPI code and the prevalence of MMI or a low MMSE score (lower panel, Figure 2).

To analyze whether the presence of fewer remaining teeth was independently related to MMI or a low MMSE score status, we calculated the multivariate-adjusted odds ratios (Table 2) using those factors with a calculated P value < 0.05 between the control and MMI groups or between the control and low MMSE score groups in Table 1. After the variables in the full models were adjusted, we observed significant relationships between the number of remaining teeth (per 1 decrease) to MMI (odds ratio: 1.021, 95% CI: 1.001-1.041) and a low MMSE score (odds ratio: 1.039, 95% CI: 1.023-1.054). Furthermore, the odds ratio of 0-10 remaining teeth to 22-32 remaining teeth was 1.679 (95% CI: 1.073-2.627) for MMI and 2.177 (95% CI: 1.510-3.140) for a low MMSE score.

**Table 2:** Odds ratios of the number of remaining teeth for MMI and low MMSE- score status

		MMI			Low MMSE score		
		Odds Ratio (95% CI)		P value	Odds Ratio (95% CI)		P value
	Per 1 decrease of remaining teeth						
Crude		1.035	(1.017 - 1.053)	<0.001	1.056	(1.042 - 1.071)	<0.001
Adjusted for depressive symptoms		--*	--	--	1.056	(1.041 - 1.070)	<0.001
Plus age, sex and length of education		1.019	(1.000 - 1.039)	0.049	1.039	(1.024 - 1.055)	<0.001
							
Adjusted for full model†		1.021	(1.001 - 1.041)	0.038	1.039	(1.023 - 1.054)	<0.001
							
	Reference: 22-32 of remaining teeth						
Adjusted for full model†	11-21	0.989	(0.601 - 1.627)	0.965	1.609	(1.099 - 2.356)	0.014
	0 -10	1.679	(1.073 - 2.627)	0.023	2.177	(1.510 - 3.140)	<0.001

MMI: mild memory impairment, MMSE: Mini-Mental State Examination, CI: confidence interval.

The number of subjects available for analysis: Control (n = 3696), MMI (n = 121), and Low MMSE score (n = 214).

*The subjects whose GDS score were "6 or more" were excluded from the MMI group.

†Full model included depressive symptoms (only for low MMSE score), age, sex, length of education, frequency of drinking, smoking habit, time spent walking every day, positive history of cancer and diabetes mellitus, and the levels of serum albumin, total cholesterol, and low-density lipoprotein cholesterol.

Among edentulous subjects (control, n = 323; MMI, n = 18; low MMSE score, n = 47), the median age (25^th ^percentile, 75^th ^percentile) at which they became edentulous and the prevalence of individuals who were edentulous for 15 years or more was 62.8 years (60.0, 68.0) and 34.4% in the control group, 62.5 years (59.8, 65.0) and 50.0% in the MMI group, and 60.0 years (57.0, 65.0) and 59.6% in the low MMSE score group, respectively, demonstrating significant differences by the Bonferroni test between the control and low MMSE score groups (data not shown). After multiple variables were adjusted (Table 3), the odds ratios of "per 1 year increase" and "15 years or more" for a low MMSE score were 1.058 (95% CI 1.014-1.103) and 3.102 (95% CI 1.432-6.720), respectively.

**Table 3:** Odds ratios of the length of the edentulous period for MMI and low MMSE-score status

		MMI			Low MMSE score		
		Odds Ratio (95% CI)		P value	Odds Ratio (95% CI)		P value
	Per 1 year increase of the period after lost all teeth						
Crude		1.040	(0.988 - 1.095)	0.133	1.063	(1.028 - 1.099)	<0.001
Adjusted for depressive symptoms		--*	--	--	1.060	(1.025 - 1.097)	<0.001
Plus age, sex and length of education		1.026	(0.967 - 1.088)	0.401	1.051	(1.010 - 1.094)	0.014
							
Adjusted for full model†		1.019	(0.960 - 1.082)	0.533	1.058	(1.014 - 1.103)	0.009
							
	Reference: The length of time after all teeth were lost <15 years						
Adjusted for full model†	15 years or more	1.279	(0.442 - 3.700)	0.650	3.102	(1.432 - 6.720)	0.004

MMI: Mild Memory Impairment, MMSE: Mini-Mental State Examination, CI: confidence interval.

The number of subjects available for analysis: Control (n = 323), MMI (n = 18), Low MMSE score (n = 47).

*The subjects whose GDS score were "6 or more" were excluded from the MMI group.

†Full model included depressive symptoms (only in low MMSE score), age, sex, length of education, frequency of drinking, smoking habit, time spent walking every day, positive history of cancer and diabetes mellitus, and the levels of serum albumin, total cholesterol, and low-density lipoprotein cholesterol.

## Discussion

This community-based survey revealed that the prevalence of a low MMSE score was significantly increased in association with the decrease in the number of remaining teeth (Figure 2). After adjustment for other explanatory variables (Table 2), a significant relationship between the decrease in the number of remaining teeth and a low MMSE score was observed. These results are consistent with those of previous reports demonstrating that tooth loss was associated with decreased cognitive function [21,22]. We also revealed that a decrease in the number of remaining teeth was associated with the risk of MMI.

Four limitations of the present study merit consideration. The principal limitation was that the data were derived from a cross-sectional study; thus, we can only hypothesize for the biological credibility of the effect of tooth loss on MMI and a low MMSE score. Second, we did not investigate when tooth loss started or its causes. We also did not investigate markers of inflammation in the gingival crevicular fluid or alveolar bone loss measurements, by which the severity of periodontal disease can be evaluated more precisely than with the CPI code. Third, our criteria for identifying subjects with MMI were different from Ishikawa's criteria [13] as we identified the absence of dementia or depression using the MMSE and GDS screening tests; however, the prevalence of MMI in our study (2.9%: 121/4,206) was similar to the prevalence of Japanese community-based mild cognitive impairment (MCI) [23,24] identified using the criteria of Petersen et al. [25]. Therefore, the MMI subjects in this study are considered to approximate individuals with MCI. Fourth, apolipoprotein E (APOE) genotyping was not examined. Individuals with dementia had a higher frequency of APOE ε4 compared with non-demented individuals in Japanese-American men [26]. The frequency of APOE ε4 was also higher in MCI subjects than in non-MCI individuals in a Japanese community [24]. These findings indicate that APOE ε4 is an important risk factor for cognitive decline. The statistical relationship between tooth loss and cognitive function will weaken relatively among subjects with APOE ε4; therefore, our findings may have overestimated the relationship between tooth loss, MMI, and a low MMSE score.

The basis of the relationship between tooth loss and a low MMSE score was considered as given below. Older adults with a low MMSE score do not regularly use dental services [27]. In addition, older adults with dementia have increased plaque accumulation [5]. There may be other biological bases separate from the deterioration of dental health induced by cognitive impairment among tooth loss, MMI, and a low MMSE score, considering that a significant relationship was also found between tooth loss and MMI subjects who maintained the basic and instrumental ADL. Four further plausible biological explanations for the relationship between tooth loss and cognitive function can be proposed. First, periodontal disease, which is the cause of approximately 50% of all extractions in the elderly [28], may be associated with cognitive function through systemic inflammation. It has been hypothesized that inflammatory cytokines induced by periodontal disease can enter or influence the brain [9,29]. Second, genetic risk factors related to periodontal disease and cognitive function may be present; an interleukin 1 gene polymorphism has been reported to be associated with the severity of periodontal disease [30] and the risk of AD [31]. Third, a decrease in the number of periodontal mechanoreceptors due to tooth loss [32], which are sensory receptors, may result in a memory learning disorder. The functional deterioration of the cholinergic neuronal system in the parietal cortex has been observed in molar-loss rats [33]. In addition, the number of high-affinity tyrosine kinase B mRNA-positive cells in the hippocampal CA3 area was negatively affected by the duration of tooth loss and the number of teeth extracted [7]. However, these findings are based on animal models, and further investigations will be needed. Fourth, other risk factors may be related to tooth loss and cognitive function. Low socioeconomic status, negative events earlier in life, head traumas with maxillofacial injuries, and limitations on the choice of a healthy diet may be related to tooth loss and cognitive function.

Among edentulous subjects, a significant relationship was found between the length of the edentulous period and the risk of a low MMSE score after adjustment for their age at the baseline examination and other variables (Table 3). A history of "lost all teeth or lost half of teeth before age 35" was a significant risk factor for AD [3]. We observed a higher prevalence of individuals who were edentulous for 15 years or more in the MMI group (50.0%) than in the control group (34.4%), although no significant relationship was found; therefore, tooth loss may have a cumulative detrimental effect on the brain.

While there was a significant relationship between the number of remaining teeth with the risk of MMI and a low MMSE score, no significant relationship was found between the CPI code, MMI, and a low MMSE score (Table 1 and Figure 2). The CPI is a marker of the current status of periodontal tissue, and it appears to underestimate the relationship between cognitive function and the cumulative burden of periodontal disease. We consider that tooth loss or periodontal disease may be associated with cognitive function because the prevalence of "Ineligible for CPI" was higher and the prevalence of "Code 0, 1, or 2" was lower in the MMI and low MMSE score groups than in the control group.

## Conclusions

Significant relationships were found between the number of remaining teeth, the length of the edentulous period, and cognitive function. Within the limitation of no APOE genotyping data, this cross-sectional study suggests a significant relationship of tooth loss to MMI and cognitive impairment. Further studies are needed to investigate the specific causes of this relationship.

## List of abbreviations

AD: Alzheimer's disease; MMI: mild memory impairment; ADL: activities of daily living; MMSE: Mini-Mental State Examination; Recall test: three-word delayed recall test; GDS: Geriatric Depression Scale; CPI: Community Periodontal Index; MCI: mild cognitive impairment; CRP: plasma C-reactive protein; CI: confidence interval;

## Competing interests

The authors declare that they have no competing interests.

## Authors' contributions

NO conducted data collection, analyses, the interpretation of data, literature review, and prepared drafts of the manuscript. MM contributed to analyses, the interpretation of the data, and the critical revision of the manuscript for important intellectual content. KO contributed to the interpretation of the data and the critical revision of the manuscript for important intellectual content. NH, JI, KT, KS, MY, and NA contributed to data collection and the critical revision of the manuscript for important intellectual content. NK was responsible for the study design and data collection, data analyses, literature review, and contributed substantially to the manuscript. All authors have read and approved the final manuscript and contributed to this work.
